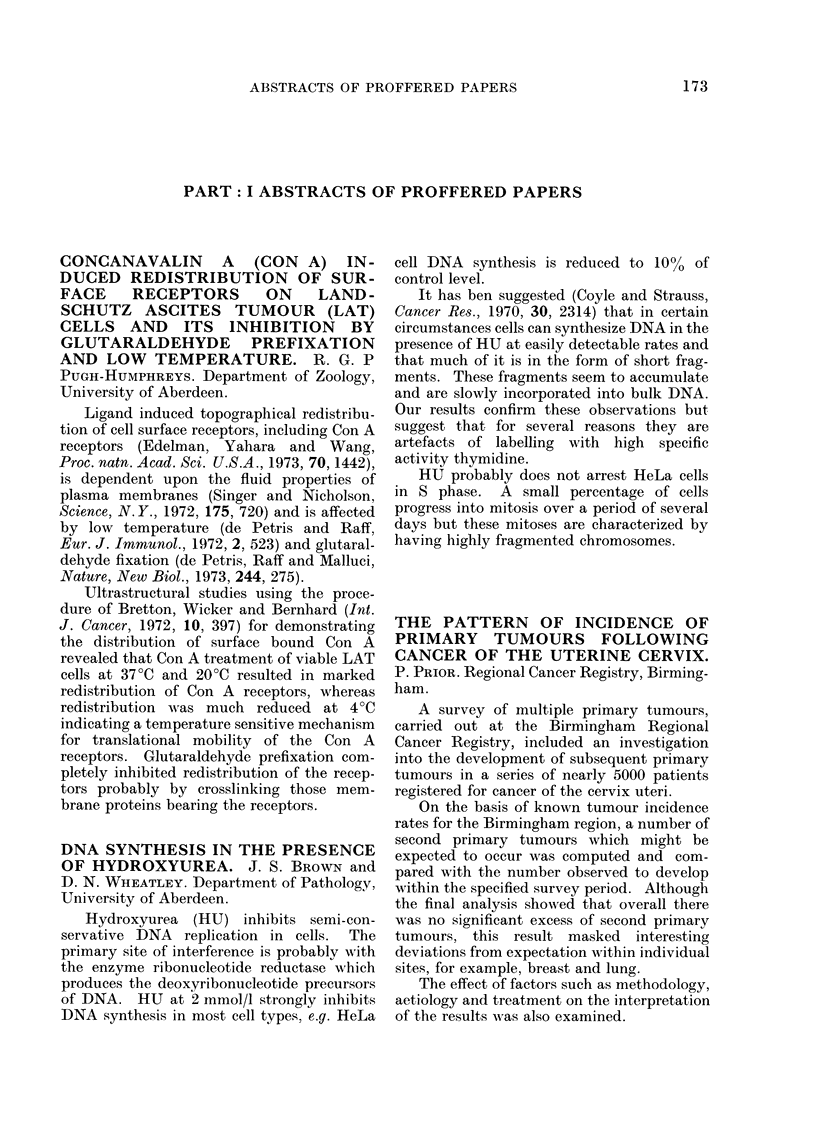# Proceedings: The pattern of incidence of primary tumours following cancer of the uterine cervix.

**DOI:** 10.1038/bjc.1974.134

**Published:** 1974-08

**Authors:** P. Prior


**THE PATTERN OF INCIDENCE OF PRIMARY TUMOURS FOLLOWING CANCER OF THE UTERINE CERVIX.** P. PRIOR. Regional Cancer Registry, Birmingham.

A survey of multiple primary tumours, carried out at the Birmingham Regional Cancer Registry, included an investigation into the development of subsequent primary tumours in a series of nearly 5000 patients registered for cancer of the cervix uteri.

On the basis of known tumour incidence rates for the Birmingham region, a number of second primary tumours which might be expected to occur was computed and compared with the number observed to develop within the specified survey period. Although the final analysis showed that overall there was no significant excess of second primary tumours, this result masked interesting deviations from expectation within individual sites, for example, breast and lung.

The effect of factors such as methodology, aetiology and treatment on the interpretation of the results was also examined.